# Assessing patient cosmetic satisfaction after glaucoma drainage device surgery for different patch grafts

**DOI:** 10.1186/s12886-021-01864-z

**Published:** 2021-02-23

**Authors:** Doaa S. Milibari, Dalal Fatani, Abeer Ahmad, Ohoud Owaidhah, Saleh A. AlObeidan, Faisal A. Almobarak, Rizwan Malik

**Affiliations:** 1 Glaucoma Division, King Khaled Eye Specialist Hospital Riyadh, Al Arubah Branch Rd, Riyadh, 11462 Saudi Arabia; 2 King Saud University Department of Ophthalmology, Riyadh, Saudi Arabia; 3 Statistics and Epidemiology, Research Department, King Khaled Eye Hospital, Riyadh, Saudi Arabia

**Keywords:** Glaucoma drainage device, Patch graft, Cosmetic satisfaction, Sclera, Pericardium, Cornea

## Abstract

**Background:**

The use of a tissue patch graft is common practice with a glaucoma drainage device (GDD). Patch grafts can be visible in the palpebral fissure and may be cosmetically displeasing for some patients. The aim of this study was to report the cosmetic satisfaction of pericardial, scleral, and corneal patch grafts related to superior GDD surgery.

**Methods:**

Baseline clinical data were collected for consecutive patients with glaucoma operated between 2014 and 2019 at two tertiary eye care institutions (for superiorly-placed) Ahmad glaucoma valve implant using sclera, cornea and pericardium patch graft. A patient questionnaire that contained 4 concise questions, with a Likert-scale grading relating to cosmetic satisfaction was administered by a telephone-based interview. Responses and scores for each question were compared across patients who received the three different types of graft. A binominal logistic regression analysis was used to assess the effects of age, gender, type of graft, number of previous ocular surgeries, and final visual acuity to explain differences.

**Results:**

We included 92 patients who met our inclusion criteria (24 patients received a corneal patch graft, 30 who received sclera and 38 who received pericardium). The mean (±SD) age was 50 (±17.5) years, and the average follow up was 20.7 (± 18.6) months. Regardless of the type of patch graft, most (67–84%) of patients were satisfied with the appearance of their eyes**.** Patients who received cornea or sclera were more likely to report that their eye looked ‘abnormal’ by others. Younger age was significantly associated with the response to this question.

**Conclusion:**

Patients are generally satisfied with the appearance of their eye following GDD surgery with each of the patch grafts for superiorly-placed GDDs. Younger patients with cornea or sclera were more likely to report that their eyes looked abnormal.

## Background

Glaucoma drainage devices (GDD) are traditionally reserved for glaucomas which are refractory to filtering surgery or in cases where trabeculectomy is at high risk for failure, although these are assuming a greater role in the primary surgical management of glaucoma [1, 2].

These devices lower the intraocular pressure by forming a space between the sclera and the conjunctiva to allow aqueous outflow. GDD an effective surgical treatment of glaucoma but is not without complications such as hypotony, tube fibrous encapsulation, plate migration, plate extrusion, corneal decompensation, strabismus, tube occlusion, tube erosion and exposure, and subsequent serious complications like endophthalmitis [2, 3].

Tube exposure and erosion of the overlying patch graft and the conjunctiva is a potentially serious complication and well-known risk factor for the development of endophthalmitis. It has been reported that 1 to 5% of eyes experience exposure of the tube through the conjunctiva within 5 years after glaucoma implant surgery. So, covering the tube with host sclera or donor patch graft is essential to prevent conjunctival erosion, and subsequent complications [4, 5].

A variety of materials have been used to cover the tube part including sclera, dura mater, glycerol-preserved cornea, gamma-irradiated cornea, partial thickness corneal graft, fascia lata, pericardium, and amniotic membrane [3, 6, 7]. Each patch graft has advantages and disadvantages in terms of clarity, surgical handling, thickness, price, cosmetic appearance, availability, and exposure rate. The ideal material for tube coverage would be immunologically safe, biocompatible, stable, cost-effective, available, easy to handle, and cosmetically acceptable [3].

Although, the exposure rates of different patch grafts have been extensively studied in the literature, largely from retrospective series [1, 2, 8, 9], the cosmetic acceptability of different patch graft has not been studied previously. Many have speculated that corneal patch grafts give cosmetically more acceptable results compared to other types of patch graft particularly as the use of a scleral patch is obvious and cosmetically displeasing to some patients [6, 8, 10–13]. However, no study has examined patient satisfaction for a different types of patch graft associated with GDD surgery and has yet to be evaluated from patient perspective. Nowadays, cosmetic surgery modalities are continuously expanding worldwide and a significant proportion of people concerned about their overall physical appearance [14, 15]. Further, there is some evidence that the cosmetic appearance of ocular tissue can affect psychological well-being and patient-reported outcomes. Therefore, the collection of evidence-based data of patient’s cosmetic satisfaction regarding the surgical outcome is essential for better health care [16].

Therefore, the present study aimed to study patient satisfaction related to the appearance of the patch graft for patients who received pericardium, sclera, or corneal patch graft related to GDD surgery.

## Methods

### Study design

We conducted a patient questionnaire of patients who underwent Ahmad valve implant surgery at King Khaled Eye Specialist Hospital and King Abdulaziz University Hospital from January 2014 and December 2019. Data were collected by chart review of medical records for all consecutive patients with glaucoma operated for Ahmad valve implant with sclera, cornea, or pericardium patch graft at two tertiary care institutions. The study was conducted between December 2018 and January 2019.

The primary outcome was to assess patient satisfaction relating to the cosmetic outcome after GDD surgery. Secondarily, we studied the rate of complications relating to each type of patch graft. The study was approved by local IRB and followed the tenants of the declaration of Helsinki.

### Surgical technique

The surgical techniques varied minimally by individual surgeon preference and detailed accounts for Ahmad valve implantation can be found elsewhere [17]. In general, a fornix-based conjunctival flap was dissected in the desired quadrant majority was superior temporal quadrant after placing a corneal 7–0 Vicryl traction suture. The Ahmed implant was primed with a balanced salt solution and the plate inserted into the exposed quadrant. The plate was sutured to the sclera using 9–0 nylon or prolene suture with the anterior edge 8–9 mm posterior to the limbus. A 23-gauge needle was used to make a limbal stab incision. The tube was trimmed to an appropriate length, and after insertion of the tube into the anterior chamber. The tube was sutured to the episclera using 9–0 nylon or prolene suture. A piece of donor patch graft (pericardium, cornea or sclera) depend on the surgeon’s preference was trimmed and sutured to the episclera to cover the tube and entry site. The corneal patch graft was lamellar dissected to achieve approximately half-thickness before use. The conjunctiva was closed with a 9–0 Vicryl sutures.

### Questionnaire development

A literature search showed that although numerous questionnaires exist to assess cosmetic satisfaction of oculoplastic lid procedures [15, 17, 18], no previous questionnaire had been developed to assess the cosmetic satisfaction of patch grafts associated with GDD surgery. As such, we developed a questionnaire specific for this purpose. The questionnaire was developed by a team of two clinicians and one epidemiologist.

The goal of the questionnaire was to assess the cosmetic satisfaction associated with the type of graft material, testing the hypothesis that corneal graft would be more pleasing to patients. The questions were developed from informal discussions with patients in the glaucoma clinic to sample the aspects of eye appearance which were important to patients. These discussions identified that patients were concerned about the appearance of their eyes in terms of comparative asymmetry, how their eyes were perceived by others and a change in appearance after surgery. The questions were, therefore, centered around these themes. In order to ascertain targeted subjective outcomes, we adopted a ‘rating-scale’ scale, consisting of 5 possible responses: ‘strongly agree’, ‘agree’, ‘neither agree or disagree’, ‘disagree’, ‘strongly disagree’. We provided a neutral response category to identify patients without a sense of satisfaction or dissatisfaction. The questionnaire evaluated the degree of ‘agreement’ or disagreement’ to five statements, which were designed to be simple, clear, concise and unambiguous. The leading question was designed to be a more general statement about eye appearance, followed by more specific questions about the type of patch graft. To maximize compliance with the questionnaire and avoid frustrating patients from lengthy questions, the questionnaire was deliberately kept short and typically took no more than 10 min to administer. The target demographic for our questionnaire included older children and adults, regardless of gender. Respondents were reassured that the questionnaire was anonymous. Demographic and surgical data was retrieved prior to the questionnaire from patients charts and was not obtained as part of the questionnaire. The questionnaire was reviewed by two Consultant Glaucoma Ophthalmologists, who were not involved in the design, to assess whether the questions were relevant and specific and this served as a form of preliminary face validity.

The questionnaire was administered by ‘phone by one of two medical professionals, who introduced themselves and verified the identity of the participant and explained the purpose of the questionnaire. It was also explained that the data was anonymous, would not affect the clinical care and the study had been granted IRB approval and they were free to refuse to take part, and would take a maximum of 10 min. The questionnaire was piloted on three patients and this pilot showed that the questions were easily understood and patients felt comfortable answering them.

The questions were asked in the native language of the region (Arabic, Saudi dialect) as not all the patients spoke English. For the purposes of reporting, a translated version to English was also back-translated to ensure that the English questions were an accurate equivalent of the Arabic version.

Briefly, we presented 4 statements as the following:

- Q1 I am satisfied with the overall cosmetic appearance of my eyes.
- Q2 Other people have not noticed the presence of the graft patch.
- Q3 Other people have commented that my two eyes look different.
- Q4 My eye looks different after surgery (compared to before surgery).

Whilst Q2 and Q3 appear similar, were designed to differentiate the perception that the patients’ eyes look different from the GDD rather than the patch itself. The answers were graded were 1 = strongly agree until 5 = strongly disagree. For statement Q1, a lower score was associated with a better cosmetic outcome; for the remaining statements, a higher score was associated with better satisfaction/appearance.

### Questionnaire administration

A telephone-based interview questionnaire was conducted by one of two investigators (DM, DF) who were blinded the type received by the patient graft. Patients receiving different types of patch graft were called in a randomized order. After assigning patients a unique study ID, a web based random number generator was used to determine an order in which to contact the patient. This was designed to minimize any interviewer bias based on the type of patch graft. The questions were asked sequentially, in Arabic, and the response recorded using the rating scale provided. The questionnaire usually took less than 5 min to administer.

### Data collection

Demographic and pre-operative, intraoperative, and post-operative data were collected. Preoperative data included patient age, gender, type of glaucoma, diabetic status, presence of surface ocular disease e.g. (chronic blepharitis, dry eye syndrome), use of anti-glaucoma drops, lens status, the total number of previous surgeries including (previous ocular surgeries that might affect the health of the conjunctiva e.g. ECCE) and pre-operative IOP reading. Intraoperative data included surgeon status, intraoperative findings relating to conjunctiva, site of the tube implantation, and type of patch graft. Post-operative data included complications, IOP measurements, and the final visual acuity at final follow-up visit.

### Inclusion criteria and exclusion criteria

To minimize confounding factors, we included only Ahmad valve GDD as we anticipated the cosmetic satisfaction might be affected by the type of GDD as well as the type of patch graft. Also, we included patients who had only superiorly placed GDD, as we anticipated that cosmesis for inferiorly placed GDD may be different from superior GDDs because of the presence of greater normal inferior scleral show in most patients. All patient with documented ptosis, proptosis, or any eyelid abnormalities which may affect their perception about eye cosmesis were excluded. Also, we excluded all patients who underwent to previous GDD, patients who underwent to other types of tubes rather than Ahmad glaucoma implant tube, any concomitant surgery such as pars plana vitrectomy, and patients who had a posterior segment GDD. We excluded all patients less than 12 years old because of the difficulties of interviewing children and we excluded all patients who did not respond after two separate attempts to contact by phone or declined to participate.

### Statistical analysis

All the data were recorded on a customized Microsoft Excel spreadsheet (Microsoft office 2007, Redmond, WA,2007). The Descriptive and inferential data analysis was performed, continuous outcomes were presented as mean (±SD) and categorical outcomes in the form of frequencies and percentages for each of the three groups. A Chi-square test was performed for categorical variables. A one-way ANOVA was used to compare means across groups for Gaussian parameters such as age and a Kruskal-Wallis test for comparing medians.

A Kruskal-Wallis test was also conducted to determine if there were differences in results for the four questions, relating to cosmetic appearance across patients receiving different patch grafts. For ease of reporting an interpretation ‘agree’ and ‘disagree’ responses were reported in these two separate categories, with the “neutral” group placed in the “agree” group. A binominal logistic regression was performed to assess the effects of age, gender, type of graft, number of previous ocular surgeries, and final visual acuity on the likelihood of score for each question when there were differences between groups. The analysis was performed using SPSS 25 (IBM Inc., Chicago, Illinois, USA). Statistical significance was set at *p* < 0.05.

## Results

One hundred and five consecutive patients who underwent to Ahmad glaucoma valve implant with different patch grafts and met the inclusion criteria were included in the study. Of these, 13 patients were excluded (11 patients did not respond to our telephone calls, one patient passed away and 1 patient was excluded due to language barriers). Of the 13 patients who did not respond to the questionnaire, their demographics of mean (±sd) age of 57.5 ± 23.2 years (95% CI for the difference: − 6.13, 20.43; t = 1.32, *p* = 0.30) and gender distribution (9 men and 4 women) were similar to the responders (Χ^2^ = 2.31, *p* = 0.13).

Thus, the study sample comprised of 92 patients: 24 patients received a corneal patch, 30 who received sclera, and 38 who received pericardium.

The mean (±SD) age was 50 (±17.5) years and the average follow up was 20.7 (± 18.6) months. Neovascular glaucoma, uveitic glaucoma, and primary open glaucoma were the main indications of GDD surgery in this study. The patients’ demographical and clinical data are summarized in Table 1**.** Group wise, the participants were youngest in the pericardium group, followed by the cornea group, then the sclera group (*p* = 0.012). There were also some differences in diagnoses across groups (*p* = 0.03), with the sclera group tending to have a higher proportion of patients with neovascular glaucoma and the cornea group having more patients with a diagnosis of congenital glaucoma. Patients in the pericardium group were also on fewer anti glaucoma medications than the other two groups (*p* < 0.01).

**Table 1:** Demographic descriptive data, (*n* = 92)

	Cornea (***n*** = 24)	Sclera (***n*** = 30)	Pericardium (***n*** = 38)	Statistic, p
**Institution**				Χ^2^ = 51.2, *p* < 0.001
1	8	28	3	
2	16	2	35	
**Age,** m ± sd	50.3 ± 18.5	57.5 ± 12.4	44.9 ± 18.9	F = 4.63, p = 0.012
**Gender (M:F)**	7: 17	18: 12	18: 20	Χ^2^ = 3.42, *p* = 0.18
**Glaucoma diagnosis**				Fe *P* = 0.03
***Open angle glaucoma***				
Primary open angle	4	3	8	
Secondary open angle	5	0	7	
***Closed angle glaucoma***				
Primary closed angle	0	3	0	
Secondary angle closure	3	3	1	
***Childhood glaucoma***				
Congenital glaucoma	3	0	1	
Juvenile glaucoma	0	0	0	
***Other glaucomas***				
Neovascular glaucoma	6	11	9	
Pseudoexfoliation glaucoma	0	3	1	
Uveitic glaucoma	2	4	8	
Aphakic glaucoma	0	1	3	
Combined mechanism	1	2	0	
**Presence of diabetes (%)**	13	16	17	Χ^2^ = 0.72, *p* = 0.70
**Number of AGM,** M (IQR)	3.0 (0.25)	3.0 (0)	1.0 (2.0)	KW Χ^2^ = 14.23, p < 0.01
**Ocular surface disease**				Χ^2^ = 1.94, *p* = 0.75
None	23	27	36	
Inflammatory conjunctival disease	0	0	0	
Exposure keratopathy	1	0	1	
Chronic blepharitis	0	3	1	
**Lens status**				Χ^2^ = 4.57, *p* = 0.33
Phakic	2	7	8	
Pseudophakic	18	22	25	
aphakic	4	1	5	
**Previous ocular surgeries**,M (IQR)	3.0 (2.0)	3.0 (1.0)	2.0 (1.0)	KW Χ^2^ = 5.47, *p* = 0.065

*M* male, *F* female, *AGM* antiglaucoma medication, *m ± sd* mean and standard deviation, *M (IQR)* median and interquartile range, *Χ*^*2*^ chi-squared statistic, *KW* Kruskal-Wallis statistic, *Fe* Fisher exact

Thirty-five (38.04%) patients had not undergone prior ocular conjunctival surgery, whilst 29 patients (31.5%) had undergone previous filtering surgery (Deep sclerectomy or trabeculectomy with or without MMC; 8 (8.70%) underwent a prior penetrating keratoplasty; 7(7.61%) underwent previous par plana vitrectomy; 9 (9.78%) had undergone transscleral cyclophotocoagulation and 4 (4.34%) had undergone other surgeries (ECCE, scleral buckle surgery). Most patients were noted to have normal conjunctival thickness during the surgery 89(97.7%); 2(2.2%) were noted to have thin conjunctiva and 1 patient was noted to have thick (1%) conjunctiva intraoperatively. There was no difference in surgeon-reported conjunctival thickness across types of patch graft (*p* = 0.119).

Most of the patients received a supratemporal tube *n* = 89 (97%), whilst the remaining 3 (3.3%) had supranasal placement. Also, there was no statistically significant difference in numbers of previous ocular surgery in relation to the different patch graft, *p* = 0.06.

The final visual acuity on last follow up at glaucoma clinic was in the range 20/20–20/70 in 26 patients (28.3%); in the range 20/80–20/160 in 15 patients (16.3%) and 20/200 – NLP in 51 patients (55.4%). There was no difference in final visual acuity across different types of patch graft (*P* = 0.540). Also, there were no significant differences in IOP measurements across patch graft groups at 3, 6- and 12-months follow-up. In 75 cases (81.5%) there were no post-operative complications of note. Early and transient complications included choroidal effusion in 3 patients (3.3%); anterior chamber shallowing in 2 patients (2.2%) and hypotony maculopathy in 1 (1%) patients which resolved without intervention. Longer-term complications included corneal decompensation secondary to tube touch in 2 patients (2.2%) and tube exposure in 1 patient (1.1%) who received initial pericardial patch graft then tube exposure was repaired using corneal patch graft. This patient was included in the ‘cornea patch’ group for questionnaire analysis.

**Fig. 1:**
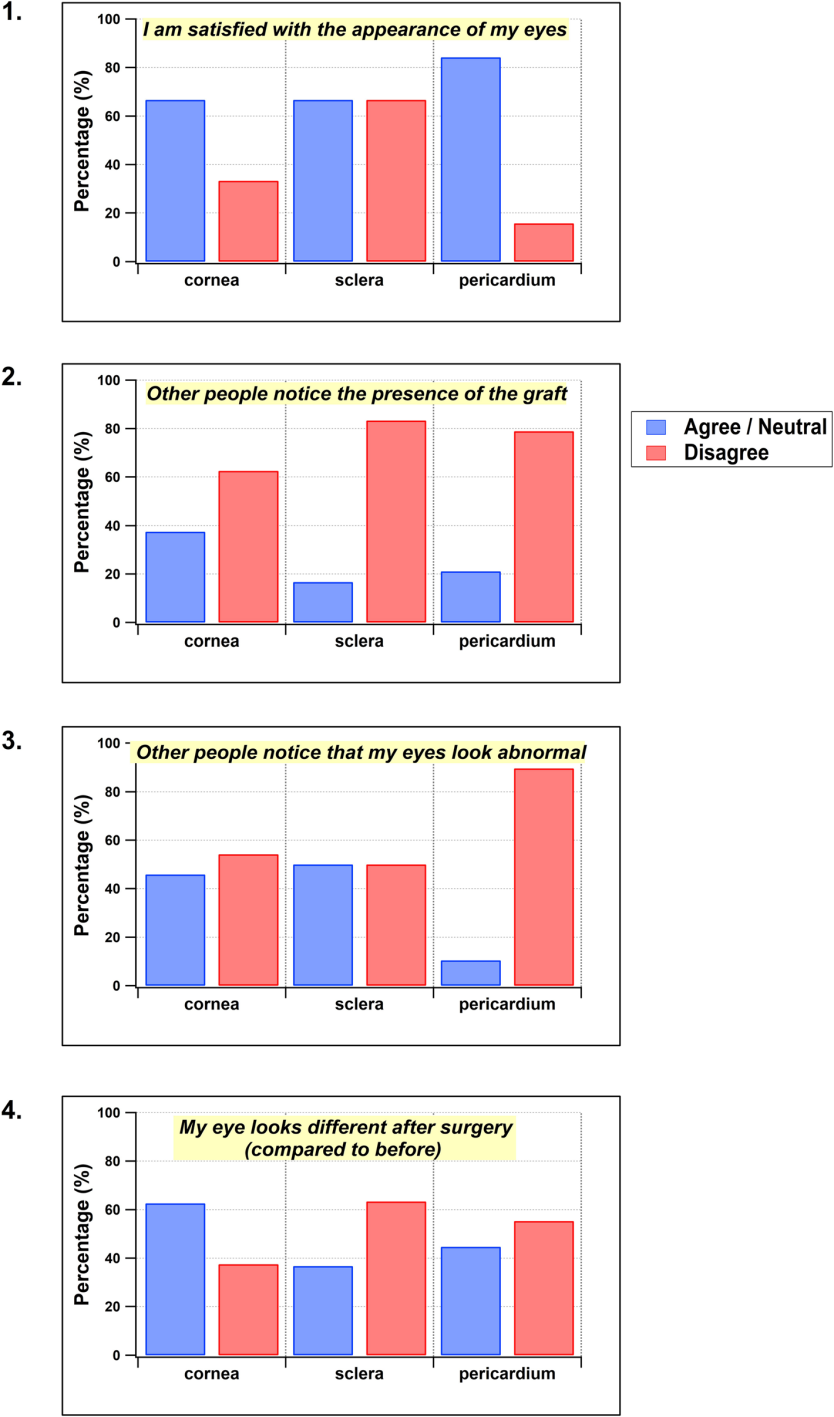
Response to questionnaire. Patients were asked whether they agreed or disagreed with one of five statements below

Figure 1 shows the results of the questionnaire of cosmetic satisfaction with each of the 3 types of patch graft. Regardless of the type of graft, most (67–84%) of patients were satisfied with the appearance of their eyes (Fig. 1a) and other people did not notice the presence of the graft in their eye (Fig. 1b).

For questions 1,2 and 4, the responses to the questions were similar across different types of patch graft (*p* = 0.18, *p* = 0.072, *p* = 0.068 respectively).

However, for statement Q3 (‘other people ask why my eyes look abnormal’), the responses were similar for patients who received cornea and scleral patch graft, with approximately half of patients agreeing with this statement; a lower proportion (10%) of patients who received pericardium felt that their eyes looked abnormal with statistically significant difference *p* = 0.009 (Fig. 1c).

To control for confounding factors and deduce baseline factors that may have contributed to differences in responses to this statement, a logistic regression was performed. Table 2 showed that age (*p* = 0.026) was a significant factor associated with an abnormal appearance, with younger patients more likely to report that their eyes looked abnormal by other people (OR = 1.038; 95% CI 1.005–1.073). The type of graft was also significantly (*p* = 0.001) associated with the response, with patients who received cornea (OR 0.089; 95% CI = 0.019─0.426) and sclera (OR = 0.053; 95% CI = 0.011─0.258) more likely to say others reported their eye as abnormal compared to patients who received pericardium.

**Table 2:** Logistic regression and factors associated with response to statement Q3 (‘Other people notice that my eye looks abnormal’)

	B	SE	Wald	df	p	OR	95% CI for OR
Age	0.037	0.017	4.971	1	*0.026	**1.038	1.005–1.073
Gender	0.183	0.550	0.110	1	0.740	1.200	0.409–3.525
Total no. surgeries	−0.093	0.217	0.184	1	0.668	0.911	0.595–1.394
Type of graft*			3929		*0.001		
Cornea	−2.421	0.800	9.149	1	*0.002	0.089	0.019–0.426
Sclera	−2.929	0.803	13.929	1	*0.001	0.053	0.011–0.258
VA:			3.143	2	0.208		
20/20–20/70	0.895	0.616	2.109	1	0.146	2.448	0.731–8.194
20/80–20/160	1.100	0.798	1.900	1	0.168	3.005	0.629–14.368
Constant	0.481	1.080	0.198	1	0.656	1.617	

Gender is for males compared to females, type of graft is for cornea and sclera compared to pericardium and visual acuity is for mild and moderate compared to poor

*OR* Odds Ratio, *VA* best-corrected visual acuity, *Wald* Wald statistic, *df* degrees of freedom

*significant results

** OR for age refers to increase with each year of age

## Discussion

It has been speculated patch graft has better cosmetic outcomes compared to the sclera or pericardium due to its translucency which, makes it cosmetically more acceptable but the current literature does not reveal any findings relating to subjective cosmetic satisfaction of different patch grafts [13].

The aim of the present study was to report and compare patient satisfaction, in terms of the cosmetic appearance of different patch grafts used for GDD surgery. We confined our study population to patients who received one type of GDD (Ahmed glaucoma valve) and had superior placement of the GDD to minimize confounding factors and allow a fair comparison across the type of graft. Given the cosmetic appearance following any kind of ocular surgery is an increasing concern for patients, and also that patients are likely to relate the success of surgery in addition to the glaucoma control.

While there is limited evidence on the effects of glaucoma surgery on perceived self-health, cosmetic surgeries in general can affect psychological and social functions such as self-esteem, overall satisfaction, mood, and social anxiety in a positive manner. Cosmesis is associated with health normality and cosmetic appearance which is perceived as abnormal can induce distress [19]. Mild degrees of facial asymmetry can be very problematic to many patients [19, 20]. Further cosmetic eyelid surgery is itself associated with satisfaction and self-esteem [15, 20]. Cosmetic satisfaction of eye appearance is likely to be associated with a better surgical outcome by patients.

The main finding from our study was that there are no marked differences in cosmetic satisfaction between pericardium, sclera, and cornea patch grafts. Superiorly-placed GDDs are usually well covered by the upper eyelid in the primary gaze and this may explain this finding. Another factor that might explain our finding is the characteristics of our study group: the majority of our patients were above 50 years of age and more than half had a severe visual loss (20/200 – NLP). Such a group may be less concerned about their eye appearance.

We had expected to find that patients with the scleral patch may be less satisfied with their cosmesis, but this was not the case. However, patients who received sclera or cornea were more likely to “agree” that other people noticed their eyes to look abnormal compared to patients who had pericardium. This suggests that others are likely to notice thicker patch grafts for superior GDDs, possibly due to a slight alteration in lid contour, as corneal and scleral patch grafts are typically much thicker than pericardium. Also, we found that younger and also female patients were more likely to report that others noticed their eyes to be abnormal post tube implant. This is an expected finding as it is well known globally women are more concerned about their appearance and bothered with slightest facial disfigurements compared to men [20, 21] Also, It has been reported that the younger age population had lower self-esteem compared to older people because they have more concerned about their appearance [20].

The results of the questionnaire demonstrated that most patients were generally satisfied with the appearance of their eyes (question 1), but also that other people did not notice the presence of the patch graft (question 2), with approximately half the patients in each group feeling that their eyes looked different after the surgery compared to before the surgery. This suggests that patients felt their eyes looked different after surgery and this may be due to the surgery itself rather than the patch graft. This finding requires further evaluation to assess the cosmetic effects of Ahmed glaucoma valve surgery itself. Ideally, this should be done prospectively, with patients questioned before and after the surgery to identify patients who may not be satisfied with ‘eye appearance’ before surgery for other reasons.

Patients with pericardium were less likely to report that their eyes looked abnormal, than patients in the cornea and sclera group, suggesting that the thinner tissue of the pericardium was more cosmetically acceptable rather than sclera or cornea. This has implications for practice, in that patients who are likely to have cosmetic concerns about superior Ahmed valve implants may be more content with a pericardial patch graft.

A number of forms of bias are relevant to this and other forms of questionnaire study, including response bias, non-response bias, prestige bias, order effects, recency bias, hostility bias, satisficing, non-differentiation bias and recall bias [22]. The most relevant forms are discussed here.

Non-response bias arises from respondents giving untruthful answers because they deem it culturally unfavorable to give negative answers. This was minimized in this study by reassuring patients that the questionnaire results would be kept anonymous and would not influence their clinical care. Non-response bias in which the patients are not representative of the intended study sample was avoided by selecting consecutive patients who had GDD surgery. Further, we demonstrated that the non-respondents had similar baseline age and gender characteristics to the responders. For responses gauged using a rating scale, non-differentiation is a possible concern, with patients giving the same answer for each question. This was handled by reversing the scale after the first question, so that patient who ‘agreed’ that he / she is satisfied with the appearance with his or her eyes would tend to ‘disagree’ that other people notice the presence of the graft.

Limitations of this study include its retrospective nature, small sample size especially corneal group, different surgeons with minimal variation in their surgical methods and surgical skills and relatively short follow up period for some patients. Also, some measure of palpebral fissure height would have been useful as cosmetic appearance is likely to be influenced by the amount of upper scleral show. However, as the lid in the normal position covers the superior sclera and none of our patients had documented lid abnormalities, it is unlikely to have affected the outcomes of this study. Another limitation of this study was the phone interview questionnaire was conducted by one of two investigators and so a sense of information bias can affect the results of the questionnaire. We minimized this by asking all questions in an objective way and standardized the method of questionnaire administration across the two interviewers.

Our questionnaire was only designed to assess five aspects of post-operative perception following GDD surgery. These questions were centered around important themes that were identified when seeing patients in clinic who had undergone GDD surgery. There are other aspects of cosmesis which were not investigated in our study e.g. more specific characteristics of the graft (color, thickness, shape, size) as well as the effect of altered lid position on perceived appearance and any psychological effects of altered appearance.

One of the main limitations of our study is that our questionnaire has not been thoroughly validated for the purpose of assessing cosmetic satisfaction of patch grafts. Although a limited face validity and pilot was conducted, a review of internal consistency was not undertaken. This would involve a prior check of cross-correlation of related questions. In addition, criterion validity, construct validity, reproducibility, longitudinal validity, responsiveness, floor and ceiling effects all should be evaluated for rigorous assessment [23].

Despite its shortcomings, this study provides some evidence that cosmetic satisfaction does not differ significantly for different types of patch grafts for superiorly-placed tubes. We expect to find such a difference for inferiorly placed patch grafts, due to the presence of greater scleral show compared to superiorly and this should form the basis of future study.

To our knowledge, this study is the first that asses the cosmetic satisfaction of different patch grafts used for GDD surgery from patient perspective.

In conclusion, we found that, overall, there was no significant difference in cosmetic satisfaction between cornea, sclera, and pericardium graft. Although there was some evidence that patients who received cornea or sclera were more likely to feel that their eye looked different compared to the pericardium. Younger age and female patients associated with less cosmetic satisfaction compared to older and men patients.

## Data Availability

Study data can be obtained on request by contacting KKESH IRB at IRB@kkesh.med.sa
